# The mediating role of depression, anxiety, and sleep in the relationship between type D personality and asthma control: a cross-sectional study

**DOI:** 10.1038/s41598-026-58701-w

**Published:** 2026-06-24

**Authors:** Şaban Melih Şimşek, Meltem Hazel Şimşek, Ruhsel Cörüt

**Affiliations:** 1 Department of Chest Diseases, Faculty of Medicine, Giresun University, Giresun, Turkey; 2 Department of Psychiatry, Faculty of Medicine, Giresun University, Giresun, Turkey

**Keywords:** Asthma, Type D personality, Asthma control, Sleep quality, Negative emotionality, Mediated analyses, Diseases, Health care, Medical research, Psychology, Psychology, Risk factors

## Abstract

Type D personality, defined by the co-occurrence of negative affectivity and social inhibition, has been associated with poorer health outcomes in chronic diseases, including asthma. However, the psychological mechanisms underlying the relationship between Type D personality and asthma control remain unclear. This cross-sectional study included 150 adult patients with asthma recruited from a tertiary outpatient clinic. Participants completed the Asthma Control Test (ACT), Type D Personality Scale (DS-14), Beck Depression Inventory (BDI), Beck Anxiety Inventory (BAI), and Pittsburgh Sleep Quality Index (PSQI). Type D personality traits were identified in 39.3% of the sample. Compared with non–Type D individuals, patients with Type D personality had higher depressive and anxiety symptom scores and poorer sleep quality (all *p* < 0.05). Mediation analyses using bootstrapping (5,000 resamples) revealed significant indirect effects of Type D personality on asthma control through depression (β = -0.088, 95% CI [-0.132, -0.047]), anxiety (β = -0.115, 95% CI [-0.158, -0.078]), and sleep quality (β = -0.057, 95% CI [-0.091, -0.029]). These indirect effects remained statistically significant after adjustment for age, sex, smoking status, and FEV1 (% predicted). These findings suggest that Type D personality is indirectly associated with poorer asthma control through increased psychological symptom burden and impaired sleep quality.

## Introduction

Type D personality is defined by the co-occurrence of two stable personality traits: negative affectivity and social inhibition. Individuals with Type D personality tend to experience increased negative emotions while simultaneously inhibiting emotional expression in social interactions. This personality construct has been associated with adverse health outcomes in various chronic diseases, particularly through heightened psychological distress and maladaptive coping patterns^1^.

Asthma is a heterogeneous chronic inflammatory airway disease characterized by variable respiratory symptoms and airflow limitation. Psychological factors are increasingly recognized as important determinants of asthma outcomes. Previous research has shown that anxiety and depressive symptoms are more prevalent among individuals with asthma and are associated with poorer disease control, reduced treatment adherence, and worse clinical prognosis^2–6^.

Psychological factors may also influence how patients perceive and report asthma symptoms. A recent review suggested that patients may underestimate or overestimate their asthma symptoms, which may subsequently affect reported asthma control scores^7^. Given that Type D personality is characterized by elevated negative affectivity and emotional distress, it is plausible that these personality traits may influence both psychological symptom burden and symptom perception in asthma.

Emerging evidence suggests that Type D personality traits may be linked to poorer asthma control. Patients with Type D personality have been reported to exhibit higher levels of depressive symptoms and negative emotionality. At the same time, anxiety and depression are common comorbidities in asthma and may influence symptom perception, disease management, and reported asthma control^4–6^. These findings raise the possibility that psychological symptom burden may represent an intermediary pathway linking personality traits to asthma outcomes.

Despite growing interest in the psychological dimensions of asthma, the potential mediating roles of depression, anxiety, and sleep quality in the relationship between Type D personality and asthma control have not been adequately investigated. Understanding these pathways may help clarify the mechanisms through which personality characteristics are associated with asthma management.

Therefore, the present study aimed to investigate the association between Type D personality and asthma control and to examine whether depressive symptoms, anxiety symptoms, and sleep quality mediate this relationship. We hypothesized that Type D personality would be associated with poorer asthma control and that this association would operate indirectly through increased depressive and anxiety symptoms and impaired sleep quality.

## Materials and methods

The present study was planned to be conducted as a descriptive cross-sectional study with asthma patients who applied to the chest diseases outpatient clinic of a tertiary university hospital in the Eastern Black Sea Region for routine control. It was hypothesized that the sample would reflect the demographics of the city, as it is one of two centers in the city where chest disease specialists are practicing. An a priori power analysis was conducted using G*Power 3.1 to estimate the required sample size for multiple regression. As no prior study was available to derive an expected effect size for the planned mediation framework, a conventional medium effect size was assumed (f² = 0.15). With an alpha level of 0.05, power of 0.80, and 8 predictors in the outcome model (Type D personality, depression, anxiety, sleep quality, and covariates), the minimum required sample size was 109 participants. The target sample size was set at 150 to account for potential missing data and to ensure adequate power. The study protocol was approved by the Scientific Research Ethics Committee of Giresun Training and Research Hospital (Approval number: 02.10.2024/01, Date: 02.10.2024). Informed consent forms were approved for all subjects included in the study. The present study was conducted in accordance with the principles outlined in the Declaration of Helsinki.

The inclusion criteria were as follows: patients who had been diagnosed with asthma by a physician, had been subjected to regular follow-up for a period of at least 1 year, were aged between 18 and 65 years, had not suffered an asthma attack in the preceding 3 months, and were receiving regular treatment for their asthma. Asthma diagnosis and clinical follow-up were established by pulmonologists according to routine clinical evaluation, including clinical history, treatment response, and spirometric assessment. FEV1 (% predicted) values obtained during routine pulmonary function testing were used as an objective indicator of pulmonary function status.

Participants with a previously diagnosed psychiatric disorder were excluded based on self-report and review of medical records. No structured psychiatric interview was conducted. However, subclinical levels of depressive and anxiety symptoms were assessed dimensionally using validated self-report scales. Participants with previously diagnosed psychiatric disorders were excluded in order to reduce the potential confounding effects of established psychiatric illnesses and psychotropic medication use on depression, anxiety, sleep quality, and asthma control. Tobacco users were excluded due to the well-established independent effects of smoking on asthma control and systemic inflammation, which could confound the mediation analyses. This approach was adopted in order to minimize the potential for confounding factors.

To minimize seasonal confounding related to environmental allergens, data collection was conducted during the period with the lowest regional pollen levels (September–October), as identified in prior regional aerobiological research^8^. Efforts were made to recruit participants from urban, district, and rural areas in proportion to the regional population distribution to reduce potential selection bias. All questionnaires were administered during the outpatient visit. The pulmonologist provided the questionnaires to eligible patients after routine clinical evaluation. Participants completed the self-report instruments independently in the clinic setting, and the completed forms were collected on the same day: the Asthma Control Test (ACT), the Type D Personality Scale (DS-14), the Beck Depression Inventory (BDI), the Beck Anxiety Inventory (BAI) and the Pittsburgh Sleep Quality Index (PSQI). Information regarding comorbid and exclusionary conditions was obtained through patient self-report during clinical history taking and verified, when available, by review of medical records.

The primary aim of this study was to examine the association between Type D personality and asthma control. The secondary aim was to investigate whether depression, anxiety, and sleep quality mediate this association.

### Asthma control test (ACT)

The Turkish version of the ACT adapted by Uysal et al. consists of five questions (Cronbach alpha: 0.84). A total ACT score is created by adding between 1 and 5 points to each question^9,10^. ACT scores range from 5 to 25, with higher scores indicating better asthma control. A score ≥ 20 is generally considered well-controlled asthma. In the present sample, the internal consistency of the ACT was assessed, and Cronbach’s alpha was 0.762.

### D-type personality scale (DS-14)

The scale under consideration consists of 14 questions and 0–4 choices in each question. It was developed by Donellet^1^, and it is comprised of two subsets: social introversion (Cronbach’s alpha: 0.86) and negative affectivity (Cronbach’s alpha: 0.88). The presence of type D personality traits is thought to be present in people with a score of 10 and above in both clusters. The validity and reliability of this questionnaire in the Turkish context was examined by Öncü et al.^11^. In the present sample, Cronbach’s alpha was 0.851 for Negative Affectivity and 0.615 for Social Inhibition. Although the internal consistency of the Social Inhibition subscale was relatively modest, similar findings have been reported in some previous DS14 studies, particularly in clinical populations^12,13^.

### Beck depression inventory (BDI)

BDI is a questionnaire comprising 21 questions designed to evaluate cognitive, emotional and motivational symptoms associated with depression. The Turkish validity and reliability of the BDI was assessed by Hisli^14^. The Cronbach alpha value for the test was determined to be 0.80.

### Beck anxiety inventory (BAI)

BAI is a 21-item questionnaire designed to assess the severity of anxiety symptoms in individuals. The Turkish version of the BAI was subjected to a validity and reliability study by Ulusoy et al.^15^, resulting in a Cronbach’s alpha value of 0.93.

BDI and BAI scores were analyzed as continuous variables. Higher scores indicate greater symptom severity.

### Pittsburgh sleep quality index (PSQI)

PSQI, a tool designed to assess sleep quality over the previous one-month period, was initially developed by Buysse et al.^16^. Turkish validation was performed by Ağargün et al.^17^. The instrument comprises seven distinct subscales. The total score is derived by summing these cluster scores. The seven clusters are as follows: sleep duration, habitual sleep efficiency, subjective sleep quality, sleep latency, sleep disturbances, use of sleeping medication, and daytime dysfunction. A total PSQI score > 5 indicates poor sleep quality.

In the present study, item-level data for the BDI, BAI, and PSQI were not retained in the database; therefore, internal consistency coefficients could not be recalculated for the current sample. We reported the previously established Turkish validity and reliability data for these instruments.

### Statistical analysis

Statistical analyses were conducted using the *SPSS Statistics 21* software. Descriptive statistics included frequency and percentage distributions, median with interquartile range, as well as mean and standard deviation values. The normality of numerical variables was assessed, and comparisons were performed accordingly: Student’s t-test was used for normally distributed variables, while the Mann-Whitney U test was applied for those not conforming to a normal distribution. Categorical variables were analyzed using the chi-square test. Additionally, correlation analyses were performed using Pearson or Spearman correlation coefficients, depending on the data distribution, to explore factors associated with asthma control. A p-value of < 0.05 was considered statistically significant.

A regression analysis based on the bootstrap method was conducted to test whether depression, anxiety, and sleep quality have a mediating role in the effect of type D personality traits on asthma disease control. Traditionally, Baron and Kenny’s methods and the Sobel test were used in mediation analyses^18^. It is claimed that the bootstrap method gives more reliable results than traditional methods and the Sobel test^19–22^. The analyses were performed using Process macro 4.2, developed by Hayes. In the analyses, the bootstrap technique with 5000 resampling options was preferred. In the mediation effect analyses conducted using the bootstrap method, the values obtained in the 95% confidence interval (CI) of the analysis results must not include the value zero (0) to support the research hypothesis^23^.

A parallel multiple mediation model (PROCESS Model 4) was tested to examine whether depression, anxiety, and sleep quality mediated the relationship between Type D personality and asthma control. To address potential confounding effects, additional mediation analyses were conducted after adjusting for age, sex, smoking status, and FEV1 (% predicted). These covariates were selected based on their potential associations with both asthma control and psychological symptoms. Indirect effects were estimated using bootstrapping with 5,000 resamples and 95% confidence intervals.

The reporting of this study was prepared in accordance with the recommendations of the ‘Strengthening the Reporting of Observational Studies in Epidemiology (STROBE)’ guideline^24^.

## Results

A total of 150 patients with asthma were included in the analysis. Of these, 39.3% met the criteria for Type D personality based on the DS-14. Baseline sociodemographic characteristics are presented in Table 1.

**Table 1:** Sociodemographic Status was presented.

Categori	Number (*n*)	%
**Age**		
Mean ± SD for Age	50,98 ± 13,91	
**Gender**	**n**	**%**
Female	124	82.7
Male	26	17.3
**Marital Status**		
Single	16	10.7
Married	115	76.7
Divorced/widow	19	12.6
**Residency**		
Urban	71	47.3
Rural	79	52.7
**Educational Status**		
Uneducated	34	22.7
Low Level	74	49.3
Middle Level	19	12.7
High Level	23	15.3
**Financial Status**		
Good	36	24
Medium	90	60
Bad	24	16
**Any Chronic Health Problem**		
Yes	94	62.7
No	56	37.3
**Smoking History (Ex-smoker)**		
Yes	26	17.3
No	124	82.7
**Family History of Any Psychiatric Diseases**		
Yes	27	18
No	123	82
**Family History of Asthma**		
Yes	105	70
No	45	30
**Asthma Medication**		
ICS	11	7.3
ICS+LABA	70	46.7
ICS+LABA+LTRA	60	40
ICS+LABA+LAMA	9	6
**Type D Personality**		
Yes	59	39.3
No	91	60.7

*ICS* Inhaled corticosteroids, *LTRA* Leukotriene receptor antagonists, *LABA* Long acting Β2 agonist, *LAMA* Long acting muscarinic antagonists.

Comparative analyses demonstrated that patients with Type D personality had significantly higher depressive symptom scores (BDI), anxiety symptom scores (BAI), and poorer sleep quality (PSQI) compared with those without Type D personality (all *p* < 0.05). Although mean PSQI scores in both groups exceeded the cut-off value of 5, indicating generally poor sleep quality among asthma patients, individuals with Type D personality demonstrated significantly worse sleep quality compared with those without Type D personality. No statistically significant difference was observed in total ACT scores between the two groups. Patients with Type D personality reported experiencing shortness of breath more frequently than non–Type D patients (Table 2).

**Table 2:** Comparison of Beck Anxiety Inventory (BAI), Beck Depression Inventory (BDI), ACT and its subtypes and Pittsburg Sleep Quality Index (PSQI) with Type D Personality Test (DS-14) were presented.

	Non-Type D Personality (*n*:91)	Type D Personality (*n*:59)	*P* Value
ACT	16.33 ± 5.50	15.39 ± 4.85	0.28
BDI	9.56 ± 7.53	16.10 ± 8.47	< 0.001 *
BAI	13.93 ± 10.15	21.88 ± 11.31	< 0.001 *
PSQI	7.47 ± 4.37	9.80 ± 5.29	0.04*
ACT 1	3.40 ± 1.41	3.00 ± 1.35	0.09
ACT 2	3.31 ± 1.48	2.80 ± 1.50	0.43*
ACT 3	3.38 ± 1.62	3.31 ± 1.63	0.77
ACT 4	2.90 ± 1.48	2.76 ± 1.47	0.57
ACT 5	3.33 ± 1.35	3.41 ± 1.40	0.73

Student’s t-test was used for group comparisons.

*Statistically significant (*p* < 0.05).

ACT total scores were negatively correlated with depressive symptoms, anxiety symptoms, sleep quality scores, number of asthma attacks in the past year, hospitalization due to asthma, and DS-14 total scores (all *p* < 0.05). No significant correlation was found between ACT scores and the social inhibition subscale of the DS-14 (Table 3).

**Table 3:** Comparison of Beck Anxiety Inventory (BAI), Beck Depression Inventory (BDI), Pittsburg Sleep Quality Index (PSQI), Number of Asthma Attacks and Hospitalisations for Asthma in the Last Year and Type D Personality Test subtypes with Asthma Control Test (ACT) and Type D Personality Test(DS-14) Scores were presented.

	Asthma Control Test ^a^	D Personality Test Scores ^a^	Negative Affect ^a^	Social Isolation ^a^
PSQI ^a^	*R*: −0.383 *P*:< 0.001	*R*:0.386 *p*:< 0.001	*R*:0.423 *p*:< 0.001	*R*:0.217 *p*:0.008
BDI ^a^	R: −0.368 P:< 0.001	R:0.580 p:< 0.001	R:0.645 p:< 0.001	R:0.314 p:< 0.001
BAI ^a^	R:−0.528 p:< 0.001	R:0.493 p:< 0.001	R:0.569 p:< 0.001	R:0.239 p:0.003
Negative Affect^a^	R:−0.322 p:< 0.001	R:0.914 p:< 0.001	*	R:0.518 p:< 0.001
Social Isolation ^a^	R: −0.008 p:0.92	R:0.821 p:< 0.001	R:0.518 p:< 0.001	*
Asthma Attacks in the Last Year ^b^	R: −0.464 p:< 0.001	R:0.106 p:0.19	R:0.190 p:0.02	R:−0.059 p:0.47
Hospitalisation for Asthma in the Last Year ^b^	R: −0.247 p:0.002	R:0.109 p:0.18	R:0.131 p:0.10	R:0.044 p:0.59
ACT Scores ^a^	*	R:−0.219 p:0.007	R:−0.322 p:< 0.001	R: −0.008 p:0.92
ACT 1 ^a^	R:0.848 p:< 0.001	R:−0.241 p:0.003	R:−0.302 p:< 0.001	R:−0.082 p:0.31
ACT 2 ^a^	R:0.743 p:< 0.001	R:−0.243 p:0.003	R:−0.315 p:< 0.001	R:−0.067 p:0.41
ACT 3 ^a^	R:0.748 p:< 0.001	R:−0.081 p:0.32	R:−0.147 p:0.72	R:0.036 p:0.66
ACT 4 ^a^	R:0.517 p:< 0.001	R:−0.094 p:0.25	R:−0.117 p:0.15	R:−0.033 p:0.68
ACT 5 ^a^	R:0.714 p:< 0.001	R:−0.179 p:0.029	R:−0.304 p:< 0.001	R:0.052 p:0.53

^a^ Pearson.

^b^ Spearman.

*p* < 0.05 was considered statistically significant in the statistical analysis.

*R* Correlation Coefficent.

Correlation analyses according to Type D personality status are presented in Table 4. Among patients with Type D personality traits, no significant correlation was observed between ACT scores and total DS-14 scores. In contrast, among patients without Type D personality traits, higher DS-14 scores were significantly associated with lower ACT scores. Negative Affectivity scores demonstrated significant negative correlations with ACT scores in the non-Type D group, whereas Social Inhibition scores were not significantly associated with asthma control in either group.

**Table 4:** Comparison of Asthma Control Test (ACT), Type D Personality Test (DS-14) and DS-14 subtypes scores between those with and without type D personality trait were presented.

	Asthma Patients with Type D Personality Traits		Asthma Patients without Type D Personality Traits	
	ACT	DS-14 Scores	ACT	DS-14 Scores
ACT	x	R:−0.097 p:0.464	x	R:−0.312 p:0.003
DS-14 Scores	R:−0.097 p:0.464	X	R:−0.312 p:0.003	X
Negative Affect	R:−0.163 p:0.219	R:0.873 p:< 0.001	R:−0.431 p:< 0.001	R:0.859 p:< 0.001
Social Isolation	R:0.023 p:0.865	R:0.784 p:< 0.001	R:0.103 p:0.332	R:0.532 p:< 0.001

*Pearson’s test was used.

*ACT* Asthma Control Test.

*p* < 0.05 was considered statistically significant in statistical analysis.

R: Correlation Coefficent.

**Fig. 1:**
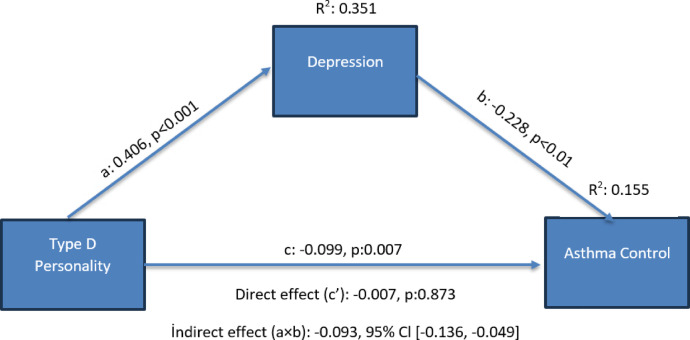
Covariate-adjusted mediation model examining the mediating role of depressive symptoms in the relationship between Type D personality and asthma control. Unstandardized beta coefficients are reported. R^2^ values represent the variance explained. Covariate-adjusted unstandardized beta coefficients are presented. Models were adjusted for age, sex, smoking status, and FEV1 (% predicted). R² values represent explained variance.

**Fig. 2:**
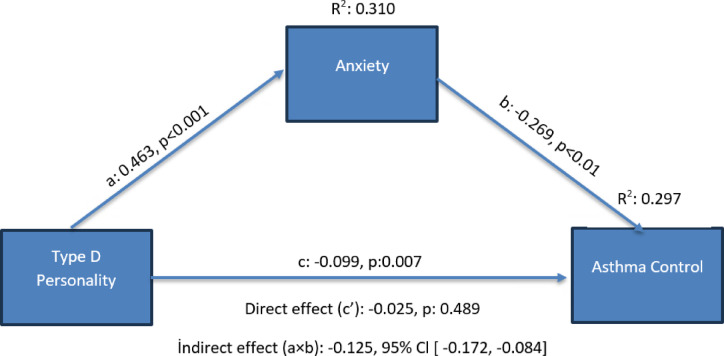
Covariate-adjusted mediation model examining the mediating role of anxiety symptoms in the relationship between Type D personality and asthma control. Unstandardized beta coefficients are reported. R^2^ values represent the variance explained. Covariate-adjusted unstandardized beta coefficients are presented. Models were adjusted for age, sex, smoking status, and FEV1 (% predicted). The mediator and outcome models explained 31.0% and 29.7% of the variance, respectively.

**Fig. 3:**
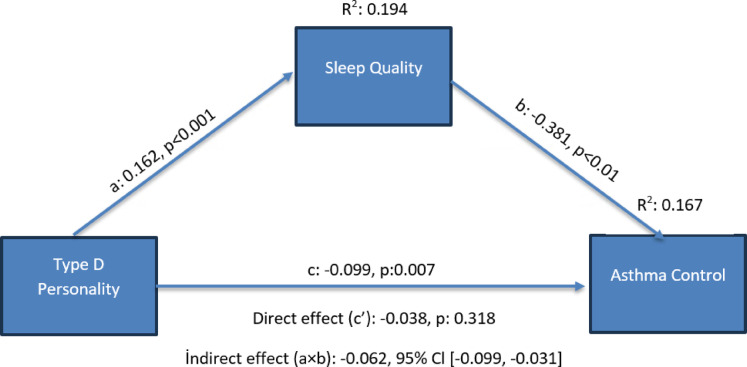
Covariate-adjusted mediation model examining the mediating role of sleep quality in the relationship between Type D personality and asthma control. Unstandardized beta coefficients are reported. R^2^ values represent the variance explained. Covariate-adjusted unstandardized beta coefficients are presented. Models were adjusted for age, sex, smoking status, and FEV1 (% predicted). The mediator and outcome models explained 19.4% and 16.7% of the variance, respectively.

The mediating effects of depression, anxiety, and sleep quality on the association between Type D personality and asthma control were examined using bootstrapped mediation analyses. Significant indirect effects were observed through depression (β = −0.088, 95% CI [−0.132, −0.047]), anxiety (β = −0.115, 95% CI [−0.158, −0.078]), and sleep quality (β = −0.057, 95% CI [−0.091, −0.029]) in the unadjusted analyses, as the 95% confidence intervals did not include zero (Figs. 1, 2 and 3).

Additional covariate-adjusted mediation analyses were conducted controlling for age, sex, smoking status, and FEV1 (% predicted). The indirect effects remained statistically significant for depression (β = −0.093, 95% CI [−0.136, −0.050]), anxiety (β = −0.125, 95% CI [−0.172, −0.084]), and sleep quality (β = −0.062, 95% CI [−0.099, −0.031]) (Table 5).

**Table 5:** Unadjusted and covariate-adjusted mediation analyses examining the indirect effects of depressive symptoms, anxiety symptoms, and sleep quality on the relationship between Type D personality and asthma control.

Mediator	Model	a path β (SE)	b path β (SE)	Direct effect c′ β (SE)	Indirect effect (95% CI)
**Depression**	**Unadjusted**	0.395	−0.225	−0.004	−0.088 [−0.132, −0.047]
**Depression**	Adjusted*	0.406	−0.228	−0.007	−0.093 [−0.136, −0.050]
**Anxiety**	**Unadjusted**	0.446	−0.259	−0.023	−0.115 [−0.158, −0.078]
**Anxiety**	Adjusted*	0.463	−0.269	0.025	−0.125 [−0.172, −0.084]
**Sleep Quality**	**Unadjusted**	0.150	−0.379	−0.035	−0.057 [−0.091, −0.029]
**Sleep Quality**	Adjusted*	0.162	−0.381	−0.038	−0.062 [−0.099, −0.031]

*Adjusted models included age, sex, smoking status, and FEV1 (% predicted) as covariates. Unstandardized beta coefficients are presented. Indirect effects were estimated using 5,000 bootstrap samples with bias-corrected 95% confidence intervals.

## Discussion

This study investigated the association between Type D personality traits and asthma control and explored the potential mediating roles of depressive symptoms, anxiety symptoms, and sleep quality. Although total ACT scores did not significantly differ between patients with and without Type D personality, mediation analyses revealed significant indirect associations through depression, anxiety, and sleep quality. These findings suggest that psychological symptom burden may represent an intermediary pathway linking Type D personality to asthma control. However, due to the cross-sectional design, the temporal and causal direction of these associations cannot be determined. Subgroup analyses demonstrated that negative affectivity was negatively correlated with ACT scores in both groups, indicating that higher levels of negative affect were associated with poorer perceived asthma control. Moreover, negative affectivity was significantly correlated with ACT items reflecting subjective symptom perception, including the frequency of shortness of breath and patient-reported asthma control. These findings suggest that emotional distress may influence how patients perceive and report asthma symptoms rather than directly affecting objective disease severity.

The prevalence of Type D personality in our sample (39.3%) was higher than rates typically reported in general population studies, where prevalence estimates are generally around 20–30%, as well as some previous asthma cohorts^1,4,5,11^. One possible explanation may be the tertiary care setting of our hospital, where patients frequently present with more complex clinical profiles and comorbid conditions. In addition, psychological distress and symptom burden may be more pronounced among patients seeking specialized outpatient care for chronic respiratory symptoms. Therefore, caution is warranted when generalizing these findings to community-based populations.

Previous studies have reported that Type D personality is associated with poorer asthma control^5,6^. For example, Witusik et al. demonstrated higher DS-14 scores among patients with poorly controlled asthma according to ACT categories^6^, and Kim et al. reported lower asthma control scores in patients with Type D personality^5^. In contrast, our study did not demonstrate a significant direct difference in total ACT scores between groups. However, the mediation analyses indicated that depressive symptoms, anxiety symptoms, and sleep disturbances statistically accounted for the association between Type D personality and asthma control. This finding may indicate that emotional distress is closely associated with the relationship between Type D personality and asthma control rather than reflecting a direct association. Our findings are consistent with those reported by Yağcı et al., who observed higher levels of anxiety, depression, and Type D personality traits among asthma patients compared with healthy controls^4^. While their study emphasized group differences, the present study extends these findings by demonstrating the potential mediating role of psychological symptoms in the association between Type D personality and asthma control.

The full mediation pattern observed in the present analyses may suggest that the association between Type D personality and asthma control is largely related to accompanying psychological symptom burden rather than a direct independent relationship. However, this does not necessarily diminish the potential clinical relevance of Type D personality. Unlike transient depressive or anxiety symptoms, Type D personality is considered a relatively stable personality construct characterized by chronic negative emotionality and social inhibition^1^. Therefore, assessment of Type D personality may still help identify individuals who are more vulnerable to psychological distress and poorer illness perception. Nevertheless, further longitudinal studies are needed to determine whether Type D personality provides incremental predictive value beyond conventional psychological symptom measures.

It may also be useful to consider the relationship between Type D personality and broader dimensional personality frameworks such as the Five-Factor Model (FFM). Negative affectivity shares conceptual similarities with neuroticism, whereas social inhibition may partially overlap with low extraversion. However, these constructs are not entirely equivalent. Interestingly, previous research examining FFM personality traits in asthma populations did not consistently demonstrate the expected associations between asthma and neuroticism alone^25^, suggesting that Type D personality may capture a more specific combination of chronic emotional distress and interpersonal inhibition. Nevertheless, because the present study did not directly assess FFM personality dimensions, these interpretations remain speculative and require further investigation.

Similar to the study by Kim et al.^5^, our research was conducted in a tertiary hospital setting. While both studies suggest a relationship between Type D personality and asthma outcomes, our study adds to the literature by applying mediation analysis to better elucidate potential psychological pathways. Future prospective studies with longitudinal designs may help clarify these associations further.

Several limitations should be acknowledged. First, the cross-sectional design precludes causal inference. Furthermore, because of the cross-sectional nature of the study, mediation analyses should be interpreted as statistical models of association rather than evidence of temporal or causal pathways. Second, all measures were based on self-report instruments, and objective markers of asthma severity (e.g., pulmonary function tests or inflammatory biomarkers) were not included. Third, the tertiary hospital setting may limit generalizability. Additionally, mediation analyses were not adjusted for potential demographic or clinical covariates, and future studies incorporating such factors may provide further clarification. Additionally, the relatively modest internal consistency observed for the Social Inhibition subscale may have reduced measurement precision and attenuated the strength of some observed associations. Psychiatric exclusion was based on self-report and available medical records, and no structured psychiatric interview was performed. Therefore, undiagnosed psychiatric disorders may have been present in some participants and could have influenced the observed psychological symptom levels and mediation analyses. Although FEV1 (% predicted) was included as an objective pulmonary function parameter, comprehensive objective markers of asthma severity, such as exacerbation frequency, GINA treatment step, eosinophil counts, FeNO measurements, or detailed longitudinal pulmonary function data, were not systematically evaluated. Therefore, residual confounding related to asthma severity may have influenced the observed associations. Finally, item-level responses for BDI, BAI, and PSQI were not retained, preventing recalculation of internal consistency coefficients in the present sample.

In conclusion, Type D personality was associated with poorer asthma control in relation to higher levels of depressive symptoms, anxiety symptoms, and impaired sleep quality. These findings highlight the potential relevance of psychological symptom burden in the comprehensive management of asthma.

## Data Availability

The datasets used and/or analysed during the current study available from the corresponding author on reasonable request.

## References

[1] Denollet, J. DS14: standard assessment of negative affectivity, social inhibition, and Type D personality. *Psychosom. Med.* **67** (1), 89–97 (2005). 15673629 10.1097/01.psy.0000149256.81953.49

[2] Barone, S. et al. The association between anxiety sensitivity and atopy in adult asthmatics. *J. Behav. Med.* **31** (4), 331–339 (2008). 18612807 10.1007/s10865-008-9164-5

[3] Lavoie, K. L. et al. Are psychiatric disorders associated with worse asthma control and quality of life in asthma patients? *Respir Med.* **99** (10), 1249–1257 (2005). 16140225 10.1016/j.rmed.2005.03.003

[4] Yağcı, İ., Perincek, G., Type, D. & Personality Somatosensory Amplification, Anxiety and Depression Symptoms Among Patients With Asthma. *Kocatepe Tıp Derg*. **25** (3), 355–360 (2024).

[5] Kim, S. R. et al. Does type personality affect symptom control and quality of life in asthma patients? *J. Clin. Nurs.* **24** (5–6), 739–748 (2015). 25257121 10.1111/jocn.12667

[6] Witusik, A. et al. Type D personality and the degree of control of bronchial asthma. *Adv. Dermatol. Allergol. Dermatol. Alergol*. **35** (4), 387–391 (2018). 10.5114/ada.2018.77670 PMC6130135 30206452

[7] Neffen, H. et al. Association and correlation of patient symptom perception and asthma control – a rapid literature review. *Asian Pac. J. Allergy Immunol.* **42** (3), 207–221 (2024). 38877849 10.12932/AP-181023-1710

[8] Ceter, T., Pinar, N. M., Turkmen, Z., Aydin, F. & Acar, A. Atmospheric pollen calendar of Giresun, Turkey. *Palynological Society of Japan*10.24524/jjpal.58.Special_31_1 (2012).

[9] Nathan, R. A. et al. Development of the asthma control test: a survey for assessing asthma control. *J. Allergy Clin. Immunol.* **113** (1), 59–65 (2004). 14713908 10.1016/j.jaci.2003.09.008

[10] Uysal, M. A. et al. The validation of the Turkish version of Asthma Control Test. *Qual. Life Res. Int. J. Qual. Life Asp Treat. Care Rehabil*. **22** (7), 1773–1779 (2013). 10.1007/s11136-012-0309-1 23143589

[11] Öncü, E. & Köksoy Vayısoğlu, S. The validity and reliability of type D personality scale in Turkish population. *Ankara Medical Journal*10.17098/amj.497485 (2018).

[12] Bunevicius, A. et al. Type D (distressed) personality and its assessment with the DS14 in Lithuanian patients with coronary artery disease. *J. Health Psychol.* **18** (9), 1242–1251 (2013). 23129829 10.1177/1359105312459098

[13] Yu, X.-n, Zhang, J. & Liu, X. Application of the type D scale (DS14) in Chinese coronary heart disease patients and healthy controls. *J. Psychosom. Res.* **65**, 595–601. 10.1016/j.jpsychores.2008.06.009 (2008). 19027450 10.1016/j.jpsychores.2008.06.009

[14] Hisli, N. A reliability and validity study of Beck Depression Inventory in a university student sample. *J. Psychol.* **7**, 3–13 (1989).

[15] Ulusoy, M., Sahin, N. H. & Erkmen, H. Turkish Version of the Beck Anxiety Inventory: Psychometric Properties. *J. Cogn. Psychother.* **12** (2), 163 (1998).

[16] Buysse, D. J., Reynolds, C. F., Monk, T. H., Berman, S. R. & Kupfer, D. J. The Pittsburgh Sleep Quality Index: a new instrument for psychiatric practice and research. *Psychiatry Res.* **28** (2), 193–213 (1989). 2748771 10.1016/0165-1781(89)90047-4

[17] Ağargün, M. Y., Kara, H. & Anlar, Ö. The validity and reliability of the Pittsburgh Sleep Quality Index. *Turk. Psikiyatri Derg*. **7** (2), 107–115 (1996).

[18] Baron, R. M. & Kenny, D. A. The moderator–mediator variable distinction in social psychological research: Conceptual, strategic, and statistical considerations. *J. Personal. Soc. Psychol.* **51** (6), 1173–1182 (1986). 10.1037//0022-3514.51.6.1173 3806354

[19] Gürbüz, S. & Bayik, M. E. A New Approach for Mediation Analysis: Is Baron and Kenny’s Method Still Valid? Türk. *Psikoloji Dergisi*. **37** (88), 15–19 (2021).

[20] Hayes, A. F. *Introduction to Mediation, Moderation, and Conditional Process Analysis: A Regression-Based Approach* Vol. 3 (The Guilford Press, 2022).

[21] Preacher, K. J., Rucker, D. D. & Hayes, A. F. Addressing Moderated Mediation Hypotheses: Theory, Methods, and Prescriptions. *Multivar. Behav. Res.* **42** (1), 185–227 (2007). 10.1080/00273170701341316 26821081

[22] Zhao, X., Lynch, J. G. & Chen, Q. Reconsidering Baron and Kenny: Myths and Truths about Mediation Analysis. *J. Consum. Res.* **37** (2), 197–206 (2010).

[23] MacKinnon, D. P., Lockwood, C. M. & Williams, J. Confidence Limits for the Indirect Effect: Distribution of the Product and Resampling Methods. *Multivar. Behav. Res.* **39** (1), 99–128 (2004). 10.1207/s15327906mbr3901_4 PMC2821115 20157642

[24] von Elm, E. et al. Strengthening the Reporting of Observational Studies in Epidemiology (STROBE) statement: guidelines for reporting observational studies. *BMJ* **335** (7624), 806–808 (2007). 17947786 10.1136/bmj.39335.541782.AD PMC2034723

[25] Najjab, A., Palka, J. M. & Brown, E. S. Personality traits and risk of lifetime asthma diagnosis. *J. Psychosom. Res.* **131**, 109961. 10.1016/j.jpsychores.2020.109961 (2020). 32105866 10.1016/j.jpsychores.2020.109961

